# Carotid arterial wall inflammation in peripheral artery disease is augmented by type 2 diabetes: a cross-sectional study

**DOI:** 10.1186/s12872-016-0397-x

**Published:** 2016-11-25

**Authors:** Sophie J. Bernelot Moens, Robert M. Stoekenbroek, Fleur M. van der Valk, Simone L. Verweij, Mark J. W. Koelemay, Hein J. Verberne, Max Nieuwdorp, Erik S. G. Stroes

**Affiliations:** 1 Department of Vascular Medicine, Academic Medical Center, Amsterdam, The Netherlands; 2 Department Vascular Surgery, Academic Medical Center, Amsterdam, The Netherlands; 3 Department of Nuclear Medicine, Academic Medical Center, Amsterdam, The Netherlands

**Keywords:** Diabetes mellitus, Imaging, Inflammation, Peripheral vascular disease, Insulin

## Abstract

**Background:**

Patients with peripheral artery disease (PAD) are at increased risk of secondary events, which is exaggerated in the presence of type 2 diabetes mellitus. Diabetes is associated with a systemic pro-inflammatory state. We therefore investigated the cumulative impact of PAD and type 2 diabetes on carotid arterial wall inflammation. As recent data suggest a detrimental role of exogenous insulin on cardiovascular disease, we also included a group of insulin users.

**Results:**

^18^F-fluorodeoxyglucose positron emission tomography with computed tomography (^18^F-FDG PET/CT) imaging showed increased carotid arterial wall inflammation, assessed as target-to-background ratio (TBR), in PAD patients without diabetes (PAD-only: *n* = 11, 1.97 ± 0.57) compared with matched controls (*n* = 12, 1.49 ± 0.57; *p* = 0.009), with a significant further TBR increase in PAD patients with type 2 diabetes (PAD-DM, *n* = 23, 2.90 ± 1, *p* = 0.033 vs PAD-only). TBR of insulin users (*n* = 12, 3.31 ± 1.14) was higher compared with patients on oral medication only (*n* = 11, 2.44 ± 0.76, *p* = 0.035), despite comparable PAD severity (Fontaine stages), BMI and CRP. Multivariate regression analysis showed that Hba1c and plasma insulin levels, but not dose of exogenous insulin, correlated with TBR.

**Conclusions:**

Concurrent diabetes significantly augments carotid arterial wall inflammation in PAD patients. A further increase in those requiring insulin was observed, which was associated with diabetes severity, rather than with the use of exogenous insulin itself.

**Electronic supplementary material:**

The online version of this article (doi:10.1186/s12872-016-0397-x) contains supplementary material, which is available to authorized users.

## Background

Although atherosclerosis is a systemic process, there is clear heterogeneity in the association between different risk factors and the vascular beds affected, implying pathophysiological differences between atherosclerotic cardiovascular disease (CVD) in different arterial regions [1]. In line, the occurrence of secondary CV events differs between patients with different primary disease manifestations: 21 % CV-events in peripheral artery disease (PAD) patients, 15 % in cerebrovascular disease patients and 15 % in coronary artery disease (CAD) patients [2]. Moreover, the event rates increase when there is more than one vascular bed affected, whereas there is a clear increase in the presence of polyvascular disease in PAD patients (60 %) compared to patients with cerebrovascular disease (15 %) or CAD (25 %). The preponderance of diffuse atherosclerotic disease and events in PAD patients has been partly attributed to a systemic pro-inflammatory state with increased c-reactive protein (CRP) levels [3, 4].

Arterial wall ^18^F-fluordeoxyglucose (^18^F-FDG) uptake has been extensively validated as a marker of atherogenic inflammation. ^18^F-FDG uptake primarily occurs in macrophage-rich areas in atherosclerotic plaques [5]. Local measurement of ^18^F-FDG is strongly associated with systemic atherosclerotic burden [6], is capable of dissecting symptomatic from asymptomatic lesions [7, 8] and finally, predicts future CV events [9]. There is only limited data on the relation between PAD and arterial wall inflammation, despite evidence of a systemic pro-inflammatory state in PAD patients and the high rate of events in other vascular territories.

Furthermore, patients with PAD and concomitant type 2 diabetes are at a two-fold increased risk of cardiovascular death compared with PAD-patients without diabetes [10]. Amongst others, this has been suggested to relate to the pro-inflammatory effects of hyperglycaemia and/or hyperinsulinemia [11, 12]. It has been speculated that exogenous insulin use could further augment systemic inflammation and subsequently increase CVD risk, based on a seemingly independent adverse effects of insulin use on cardiovascular risk [13], combined with inflammatory actions of insulin in vitro [14]. However, there is currently no direct evidence that increased systemic arterial wall inflammation may be implicated in the cumulative impact on cardiovascular risk of PAD and type 2 diabetes, nor has the association between exogenous insulin use and arterial wall inflammation been previously investigated.

The primary objective of our study is to determine whether: i) the high burden of polyvascular disease and systemic inflammation in PAD is reflected in increased inflammatory activity of the carotid arterial wall and ii) if concomitant type 2 diabetes augments arterial wall inflammation. We also aimed to explore whether such inflammatory activity is further influenced by insulin-use. Therefore, we assessed carotid arterial wall inflammation in PAD patients with or without type 2 diabetes. Subsequently, we compared ^18^F-FDG uptake between PAD patients with type 2 diabetes with or without exogenous insulin in addition to oral antidiabetic drugs.

## Methods

### Study design and population

We performed a cross-sectional study in a random selection of patients with PAD who visited the outpatient clinic between August 2014 and March 2015. PAD was defined as an Ankle-Brachial Index (ABI) <0.9 and/or a decrease of >0.15 in ABI after a treadmill-test, and the presence of exertional leg pain or rest pain [15]. Exclusion criteria were type 1 diabetes, presence of tissue loss (Fontaine stage IV), history of a recent CV-event (i.e., myocardial infarction or cerebrovascular event within the last 6 months) and clinical or biochemical signs of acute infection (fever or CRP >10 mg/dl). Subjects were divided in three groups: 1) PAD without diabetes (PAD-only), 2) PAD with type 2 diabetes using oral antidiabetic drugs only (NIDDM) or 3) PAD using a combination of oral antidiabetic drugs and insulin (IDDM). Groups were matched for age, gender, body mass index (BMI) and smoking. The study was conducted at the Academic Medical Center in Amsterdam, the Netherlands. The study protocol was approved by the institutional review board of the Academic Medical Center in Amsterdam, The Netherlands. Written informed consent was obtained from each participant.

### Medical history, biometric and biochemical measurements

All subjects underwent cardiovascular risk assessment including risk factors and family history. Medication use and Fontaine classification [15] were extracted from the medical history. Physical examination, including weight, height and blood pressure was performed. Basal fasting glucose, HbA1c, total cholesterol, HDL and LDL cholesterol, triglycerides and free fatty acids (FFAs) were assessed in fasting plasma using standard laboratory procedures. Glucose was determined using finger stick blood glucose measurements, insulin was determined on an Immulite 2000 system (Diagnostic Products, Los Angeles, CA). C-peptide was measured by RIA (RIA-coat C-peptide; Byk-Sangtec Diagnostica, Dietzenbach, Germany). Homeostasis model assessment (HOMA) index was calculated as a measure for insulin sensitivity (HOMA = insulin (pmol)/6.945*glucose (mmol)/22.5).

### ^18^F-FDG- PET/CT Imaging

^18^F-FDG positron emission tomography with computed tomography (PET/CT) scans of the carotid arteries were performed on a dedicated scanner (Philips, Best, the Netherlands). Subjects fasted for at least 6 h before infusion of 100 MBq ^18^F-FDG. The PET scan was performed 90 min post-infusion, with a low-dose, non-contrast enhanced CT for attenuation correction and anatomic co-registration (slice thickness 3 mm). The scan started at the meatus acusticus externa and ended at the caudal side of the heart, including 3 bed positions. Because of ethical constraints relating to radiation exposure, age and gender matched healthy controls were selected from a contemporaneous study using identical imaging protocols and performed on the same scanner.

### Image analysis

Images were analyzed with dedicated software (OsiriX, Geneva, Switzerland; http://www.osirix-viewer.com) by experienced readers blinded for patient data (SLV, FvdV, intra- and inter-observer agreement was excellent for FDG uptake metrics in the arterial wall, with ICCs of >0.94 with narrow 95 % confidence intervals [16]). In the carotid arteries, ^18^F-FDG uptake was assessed in at least five regions of interest (ROI). From each ROI, the mean and maximum standardized uptake values (SUVs) were obtained. The SUV represents the ^18^F-FDG activity in the ROI (in kBq/ml), adjusted for the administered ^18^F-FDG dose, corrected for decay (in MBq) and divided by body weight (in kg). Mean and maximum SUVs were averaged for each arterial segment (SUV_mean_ and SUV_max_). As previous studies have shown that hyperglycemia lowers ^18^F-FDG uptake [17], SUV values were corrected for fasting pre-scan glucose levels as described previously, normalizing measured glucose levels for an overall population average of 5 mmol/L [11]. Background ^18^F-FDG activity was measured in the venous blood pool as an average of at least 5 ROIs.

The glucose-corrected TBR was obtained for both carotids by normalizing the respective glucose-corrected SUV values in the arteries for blood pool activity, resulting in TBR_mean_ and TBR_max_. Three adjoining segments with the highest TBR were used to calculate the most diseased segment (TBR_mds_). For each subject, the artery with the highest glucose corrected TBR (index vessel) was used for comparisons.

### Statistical analysis

The sample size calculation was guided by previously reported differences in mean maximal TBR between CVD patients and healthy controls, because insufficient evidence exists regarding the potential cumulative effect of PAD and type 2 diabetes. The calculations (which are provided in the Additional file 1: Table S1) yielded a sample size of 17, and we chose to increase the number to approximately 30 participants because we anticipated smaller differences between PAD patients with or without diabetes versus PAD patients and healthy controls. All data were analysed using Prism version 5.0 (GraphPad software, LaJolla, California) and SPSS version 21.0 (SPSS Inc., Chicago, Illinois). Continuous variables are expressed as means and standard deviations (SD) or median and interquartile range (IQR), unless otherwise specified. Differences in baseline characteristics between the groups were assessed using t-tests or Mann Whitney *U* test depending on distribution for continuous variables, and Chi-square tests for categorical variables. Differences in TBR between controls and PAD-only patients, PAD-DM and PAD-only patients, as well as between PAD-IDDM and PAD-NIDDM, were assessed with a univariate analysis of covariance (ANCOVA) to account for age and gender.

### Multiple linear regression

Multiple linear regression analysis with backward elimination was used to disentangle the relative contribution of cardiovascular risk factors and factors related to diabetes to the various glucose-corrected TBR parameters, both in the overall group and in the subgroup of patients with diabetes. The initial model included age, gender, BMI, smoking, systolic blood pressure, TC/HDL-ratio, CRP and diabetes. In the secondary analysis, which aimed to explore whether exogenous insulin use independently contributed to the study outcomes HbA1c and fasting circulating insulin were additionally included as measures of diabetes regulation. As HbA1c and circulating insulin were non-normally distributed (Shapiro-Wilk *p* = 0.020 and *p* = 0.015 respectively), values were log transformed before entering them into the model. Backward stepwise elimination (probability for removal *p* > 0.1) resulted in the final model, and variables with a *p* < 0.05 were considered statistically significant.

### Exploration of associated variables

Pearson or Spearman’s rho correlation coefficients (depending on normal distribution) were calculated to assess multi-collinearity between explanatory variables. To provide additional support for any observed relations between diabetes- and insulin-related variables and arterial wall inflammation, Pearson or Spearman’s rho correlation coefficients (r) were calculated for both glucose and HbA1c, as well as between c-peptide and exogenous insulin dose and various TBR-measures and various TBR measures.

## Results

### Baseline characteristics

Baseline characteristics are summarized in Table 1. We included 34 PAD patients (age 64 ± 7), 11 of whom had PAD only (PAD, age 66 ± 6), and 23 had PAD and diabetes (PAD-DM, age 63 ± 7) and 11 age and gender matched controls (control, age 63 ± 3). The majority of the PAD patients had Fontaine stage 2 (a or b). PAD severity was equally distributed over diabetic and non-diabetic patients and did not differ between insulin and non-insulin users (Additional file 1: Table S2). The PAD-DM group consisted of 11 patients using oral antidiabetic drugs only (PAD-NIDDM, age 64 ± 7) and 12 patients also using insulin (PAD-IDDM, age 63 ± 7). As expected, PAD patients had a worse CV risk profile compared to controls, with higher BMI, blood pressure and more smokers as well as increased CRP levels. Most PAD subjects used a statin, and statin use was not different between subjects with and without diabetes. Anti-hypertensive medication was more frequently used in subjects with diabetes (medication use is specified in Additional file 1: Table S3). All PAD subgroups were matched for gender Systolic blood pressure, lipid profile and circulatory markers of inflammation (CRP, leucocyte count) did not differ between PAD subgroups. As expected, fasting glucose and HbA1c levels were higher in the diabetic group (glucose; PAD only: 5.7 ± 0.4, PAD, DM: 8.0 ± 2.6, *p* = 0.001. HbA1c; PAD only: 42 ± 6, PAD, DM: 62 ± 18, *p* < 0.001). Within the diabetic subjects, HbA1c and HOMA-IR was significantly higher in insulin dependent subjects (Hba1c; NIDDM: 51 ± 16, IDDM: 71 ± 15, *p* = 0.006. HOMA-IR; NIDDM: 3,2[2,9-5,4], IDDM 9,8[5,0-16], *p* = 0.038). C-peptide was significantly lower in patients using exogenous insulin.

**Table 1:** Clinical Characteristics of Controls and Peripheral artery disease (PAD) patients, with and without type 2 Diabetes Mellitus (DM)

	Control *n* = 11	PAD all *n* = 34	*P* value	PAD only *n* = 11	PAD-DM *n* = 23	*P* value PAD vs PAD-DM	PAD-NIDDM *n* = 11	PAD-IDDM *n* = 12	*P* value NIDDM vs IDDM
Age, years	63 ± 3	64 ± 7	0.322	66 ± 6	63 ± 7	0.243	64 ± 7	63 ± 7	0.781
Gender,%male (n)	64 (7)	64 (21)	0.949	64 (7)	61 (14)	0,616	55 (6)	67 (8)	0.735
BMI, Kg/m^2^	26 ± 2	29 ± 4	<0.001	29 ± 4	29 ± 4	0,752	30 ± 5	29 ± 3	0.640
Smoking, % active	25 (3)	46 (15)	<0.001	50 (5)	44 (10)	0.912	45 (5)	35 (6)	0.692
SBP	136 ± 6	155 ± 19	<0.001	151 ± 16	156 ± 21	0.436	151 ± 19	161 ± 22	0.281
DBP	81 ± 4	81 ± 10	0.886	82 ± 11	81 ± 9	0.785	82 ± 6	80 ± 11	0.526
TotChol, mmol/L	5.7 ± 0.8	4.4 ± 0.9	<0.001	4.8 ± 0.8	4.3 ± 0.9	0.097	4.2 ± 1.3	4.3 ± 0.4	0.807
LDL, mmol/L	3.7 ± 0.6	2.5 ± 0.8	<0.001	2.9 ± 0.7	2.4 ± 0.8	0.109	2.4 ± 1.1	2.4 ± 0.5	0.952
HDL, mmol/L	1.4 ± 0.4	1.2 ± 0.4	0.116	1.4 ± 0.4	1.2 ± 0.3	0.144	1.2 ± 0.3	1.2 ± 0.4	0.102
TG, mmol/L	1.1 [0.7–1.5]	1.3 [0.9–2.0]	0.196	1.1 [0.8–2.1]	1.3 [1.0–2.1]	0.384	1.2 [1.1–1.6]	1.6 [0.9–2.6]	0.525
CRP, mg/dl	1.3 [0.9–2.1]	2.6 [1.4–5.7]	0.038	1.8 [1.4–2.7]	2.9 [1.4–6.4]	0.144	2.9 [1.9–8.1]	2.9 [0.7–6.2]	0.525
Leucocytes, 10^9^/L	6.0 ± 1.4	7.4 ± 2.5	0.083	6.8 ± 2.2	7.7 ± 2.6	0.370	8.0 ± 2.4	7.6 ± 2.5	0.624
Diabetes	n/a	n/a	n/a	No	Yes	n/a	Yes	Yes	n/a
Glucose, mmol/L	5.3 ± 0.4	7.3 ± 2.5	<0.001	5.7 ± 0.4	8.0 ± 2.6	0.001	7.1 ± 1.4	8.9 ± 3.2	0.102
Insulin, pmol/L	n/a	n/a	n/a	89 [49–175]	103 [67–178]	0.464	85 [60–140]	174 [78–300]	0.122
Hba1C									
*mmol/mol*	n/a	n/a	n/a	41 [38–49]	57 [48–82]	<0.001	49 [43–57]	78 [57–85]	0.006
*%*	n/a	n/a	n/a	5.9 [5.6–6.6]	7.4 [6.5–9.7]		6.6 [6.1–7.4]	9.3 [7.4–9.9]	
c-peptide, mmol/L	n/a	n/a	n/a	560 [420–970]	670 [550–880]	0.621	800 [660–1110]	565 [355–790]	0.032
HOMA-IR	n/a	n/a	n/a	2.3 [1.3–6.0]	5.1 [3.0–10.4]	0.032	3.2 [2.9–5.4]	9.8 [5.0–16]	0.038

Values are % (n), mean ± SD or median [IQR,] for skewed data. PAD indicates Peripheral artery disease. DM (type 2 diabetes mellitus). *NIDDM* non-insulin dependent diabetes mellitus, *IDDM* insulin-dependent diabetes mellitus, *BMI* body mass index, *SBP* systolic blood pressure, *SDP* diastolic blood pressure, *TotChol* total cholesterol, *LDL* low density lipoprotein, *HDL* high density lipoprotein, *TG* triglycerides, *CRP* c-reactive protein, *HOMA-IR* homeostatic model of insulin resistance

### Arterial wall inflammation

**Fig. 1:**
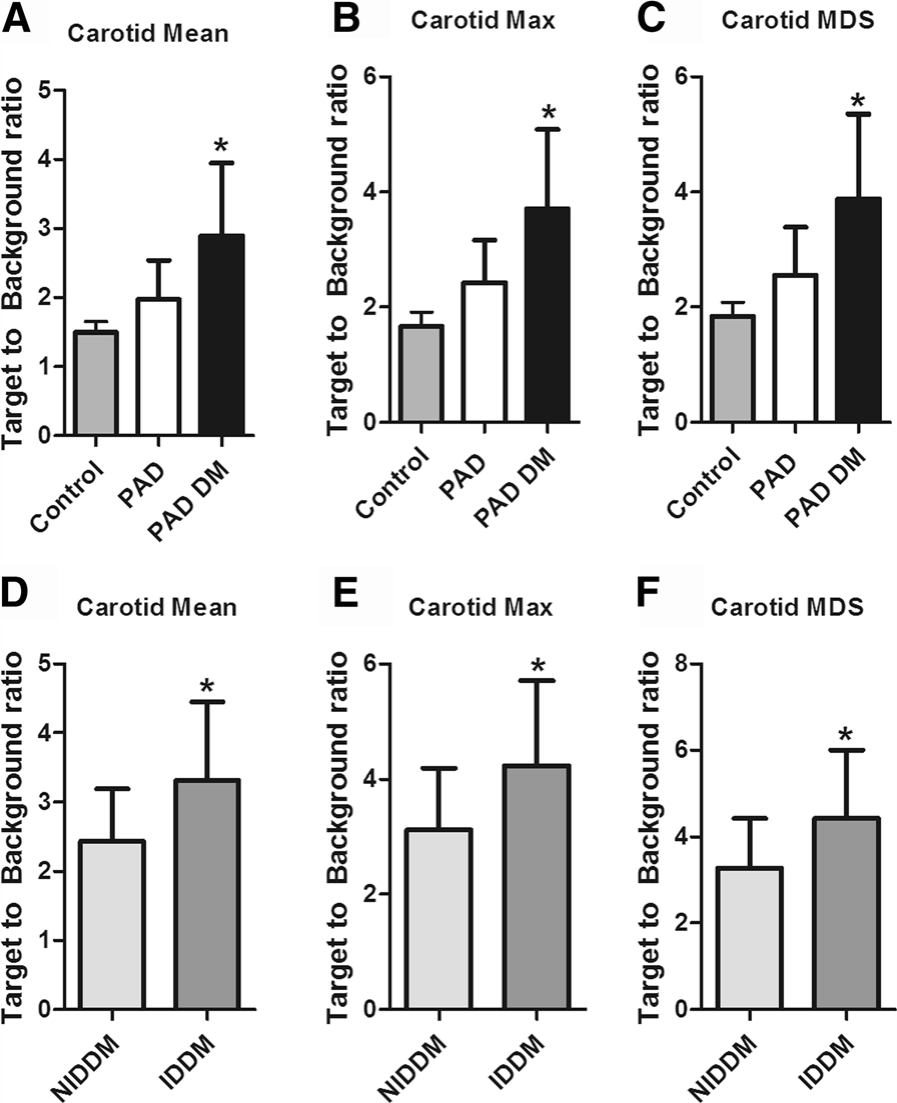
Quantification of ^18^F-FDG uptake as the target to background ratio (TBR) in the whole (**a**, **d**) carotid arteries, as well as the maximum TBR (**b**, **e**) and TBR in the most diseased segment (MDS) (**c**, **f**) revealed increased uptake in all segments in PAD subjects compared with healthy controls, and a further increase in PAD subjects with concomitant Diabetes Mellitus (**a-c**). Within Diabetic subjects, insulin dependent subjects (IDDM) had more severe arterial wall inflammation compared with non-insulin dependent subjects (NIDDM) (**d-f**). *P* values are adjusted for age and gender. *p < 0.05, **p < 0.01, ***p < 0.001

Overall, we found significantly elevated ^18^F-FDG uptake in PAD patients compared to controls (TBR_mean_ PAD 2.60 ± 1.01 vs Control 1.49 ± 0.57, *p* = 0.001 data not shown). In non-diabetic PAD patients, TBR_mean_ was 1.97 ± 0.57, which was still significantly elevated compared with the non-PAD control cohort (*p* = 0.009 (ANCOVA adjusted for age and gender). Compared with non-diabetic PAD patients, diabetic PAD patients showed an almost 1.5 fold increase in ^18^F-FDG uptake (PAD-DM; TBR_mean_: 2.90 ± 1.05, *p* = 0.033 adjusted for age and gender). Maximum carotid uptake and uptake in the Most Diseased Segment (MDS) showed similar differences (Fig. 1 a-c). Within the diabetic patients, the increase in TBR was most pronounced in insulin dependent subjects (PAD-IDDM; TBR_mean_: 3.31 ± 1.14 versus PAD-NIDDM; 2.44 ± 0.76, *p* = 0.035), with similar results for TBR_max_ and TBR_mds_ (Fig. 1 d-f).

### Multiple regression analysis

As the PAD cohort had a significantly worse CV risk profile and elevated CRP levels compared to controls, we set out to investigate which factors contributed to the difference in TBR. In the complete cohort of PAD patients, the presence of diabetes was the only predictor which remained significantly correlated with glucose-corrected carotid ^18^F-FDG uptake after stepwise backward elimination (Table 2). BMI and systolic blood pressure reached the *p* = 0.10 threshold and were retained in the model, but did not show a significant correlation with TBR (Table 2).

**Table 2:** Multiple Linear Regression Analyses with Backward Elimination to Identify Markers that Predict Carotid Arterial Wall inflammation in PAD patients

	Standardized coefficient β	95 % confidence interval	*p-value*
Glucose-Corrected TBR_mean_			
Diabetes	0.357	0.068–1.455	0.033
BMI	0.320	−0.001–0.169	0.053
Systolic Blood Pressure	0.312	−0.001–0.033	0.062
Glucose-Corrected TBR_max_			
Diabetes	0.382	0.160–2.006	0.023
BMI	0.311	−0.005–0.221	0.060
Systolic Blood Pressure	0.285	−0.003–0.042	0.086
Glucose-Corrected TBR_mds_			
Diabetes	0.368	0.117–2.104	0.030
BMI	0.304	−0.009–0.234	0.068
Systolic Blood Pressure	0.285	−0.004–0.045	0.090

Whole Carotid glucose Corrected Target-to-Background ratio (TBR_mean_), maximum uptake (_TBRmax) and_ Most Diseased Segment (TBR_mds_) were the response variables. Explanatory factors comprised Cardiovascular risk factors (age, gender, BMI, smoking, systolic blood pressure, TC/HDL-ratio, CRP and diabetes) and the inflammatory marker CRP. Variables were retained in the model when *P* < 0.10. Data are standardized coefficient (β) with 95 % confidence intervals (CI)

### Multiple regression analysis in the diabetic subgroup

To disentangle the association between exogenous insulin use and disease severity on the one hand, and arterial wall inflammation on the other, we performed multiple regression analysis in the PAD-DM group, adding circulating insulin and Hba1c as explanatory variables. Within this group, Hba1c and circulating levels of insulin were significantly correlated with whole carotid ^18^F-FDG uptake (Table 3).

**Table 3:** Multiple Linear Regression Analyses with Backward Elimination to Identify Markers that Predict Carotid Arterial Wall inflammation in PAD patients with concomitant Diabetes

	Standardized coefficient β	95 % confidence interval	*p-value*
Glucose-Corrected TBR_mean_			
Circulating Insulin	0.541	0.514–1.458	0.001
Hba1c	0.537	1.014–2.903	0.001
Glucose-Corrected TBR_max_			
Circulating Insulin	0.552	0.550–2.049	0.002
Hba1c	0.477	0.748–3.748	0.006
Glucose-Corrected TBR_mds_			
Circulating Insulin	0.536	0.723–4.007	0.008
Hba1c	0.480	0.501–2.142	0.004

Whole Carotid glucose Corrected Target-to-Background ratio (TBR_mean_), maximum uptake (TBR_max) and_ Most Diseased Segment (TBR_mds_) were the response variables. Explanatory factors comprised cardiovascular risk factors (Age, Gender, BMI, smoking, Systolic Blood Pressure) and factors associated with Diabetes regulation (Insulin and Hba1c, both log-transformed). Variables were retained in the model when *P* < 0.10. Data are standardized coefficient (β) with 95 % confidence intervals (CI)

### Exploration of associated variables

**Fig. 2:**
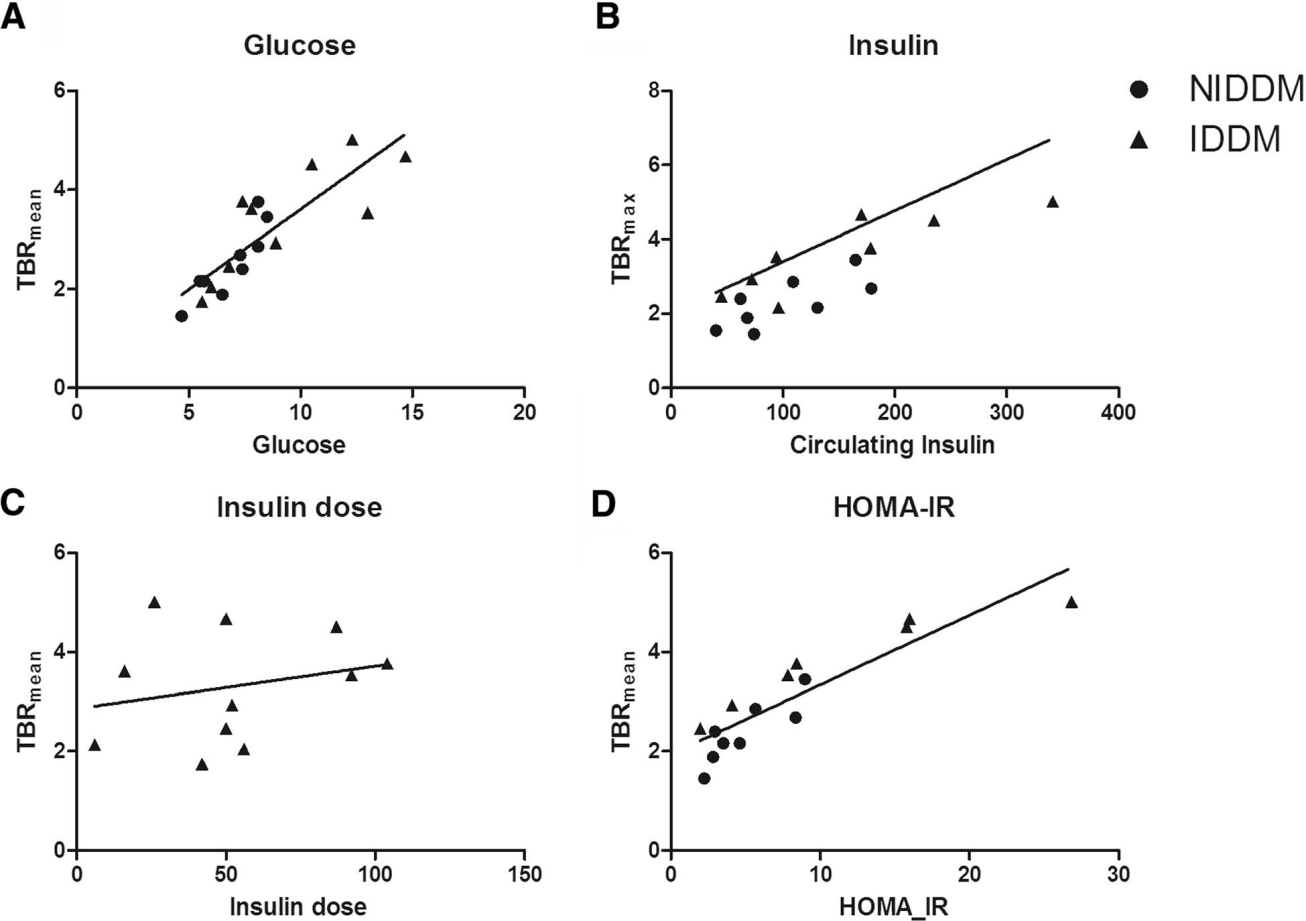
Target to Background ratio (TBR_mean_) significantly correlated to fasting glucose levels (Correlation coefficient: 0.845, *p* < 0.001) (**a**). Fasting insulin correlated significantly to TBR_mean_ (**b**) but dose of exogenous insulin did not (Insulin: Correlation coefficient: 0.814, *p* < 0.001; Exogenous insulin dose: Correlation coefficient: 0.235, *p* = 0.486). Insulin resistance, measured by HOMA-IR, stongly associated with TBR_mean_ (Correlation coefficient:, 0.876, *p* < 0.001)

Next, we assessed factors influencing Hba1c and Insulin (which did not correlate to each other (*r* = 0.217, *p* = 0.387)). As expected, fasting glucose and Hba1c showed significant correlation to each other (r: 0.601, *p* = 0.004), and following, fasting glucose showed significant correlation to carotid arterial wall inflammation (r: 0.845, *p* < 0.001) (Fig. 2a). On the other hand, although fasting insulin levels correlated with arterial wall inflammation (Fig. 2b), neither c-peptide levels nor dose of exogenous insulin were associated with circulating insulin levels (Additional file 1: Table S4) or with carotid ^18^F-FDG uptake (Insulin dose: Fig. 2c, C-peptide: Additional file 1: Figure S1). This raised the suggestion that association of the circulating levels of insulin with arterial FDG uptake is a function of insulin resistance, which was corroborated by a strong correlation between HOMA-IR and arterial wall inflammation (Fig. 2d).

## Discussion

In the present study, we demonstrate that PAD patients have elevated carotid arterial ^18^F-FDG uptake, substantiating the presence of a systemic pro-inflammatory state in these patients. Concomitant diabetes further augments the arterial wall inflammation 1.5-fold. Previous studies led to speculations on a possible independent effect of insulin use on cardiovascular risk. However although we did find increased carotid ^18^F-FDG uptake among insulin-users, this can probably not be explained by direct effects of exogenous insulin use, since ^18^F-FDG-uptake was only positively associated with HbA1c and total plasma insulin concentration but not with exogenous insulin dose. These data imply a role for hyperglycaemia and/or insulin resistance rather than exogenous insulin use.

### PAD, inflammation and secondary events

Our results support the concept that increased systemic arterial wall inflammation mechanistically links PAD with the preponderance towards secondary cardiovascular events [2, 18, 19]. Previous studies showed that increased CRP levels are associated with an increased CVD risk in PAD subjects [20–22]. Nevertheless, it has been shown that CRP alone cannot explain future CV morbidity and mortality in PAD patients [23, 24], indicating that it is merely a marker and not a causal factor in CVD risk. More recently, local inflammatory activity of the arterial wall, assessed with ^18^F-FDG uptake, emerged as a predictive marker for future CV-events [9]. FDG arterial wall uptake occurs predominantly in macrophage rich areas [7], correlates with plaque macrophage content [8] and is associated with active culprit lesions [7, 25]. As such, our finding that PAD patients have significantly increased TBR in the carotid arteries compared with age and gender matched controls, suggests that arterial wall inflammation may be involved in the high burden of polyvascular disease in PAD patients, emphasized by the fact that coronary events are the major cause of death in this group [2]. In line with previous studies [8, 9], in our multivariate model, CRP levels did not influence inflammatory activity of the arterial wall, implying that CRP levels do not directly influence arterial wall inflammation in PAD patients.

### Factors influencing arterial inflammation in PAD patients

Our finding that PAD and type 2 diabetes have an additive effect on carotid arterial wall inflammation suggests that systemic arterial wall inflammation may be implicated in the particularly elevated cardiovascular risk of patients with this combination. Previous studies reported increased arterial inflammation in patients with type 2 diabetes [11]. One possible explanation for our finding is that PAD patients with concomitant diabetes have more advanced PAD. However, clinical PAD disease severity assessed with the Fontaine classification did not significantly differ between the groups. ABI is of limited value in comparing PAD severity, because diabetes is known to falsely elevate ABI values [15]. Several factors other than PAD disease severity may contribute to the increased inflammation in patients with PAD and concomitant type 2 diabetes. The impact of hyperglycaemia and insulin resistance on arterial inflammation in humans has been corroborated using PET/CT as surrogate marker [11, 12]. However, data on glucose homeostasis from these studies are limited to a relation between hyperglycemia and ^18^F-FDG uptake, and these studies comprised only type 2 diabetes patients who used oral antidiabetic medication. We elaborated on these findings, by including HbA1c and (fasting) insulin levels in our analyses. In a multivariate model, both circulating insulin and HbA1c were associated with increased ^18^F-FDG uptake, proposing a strong association between diabetic control and arterial wall inflammation.

In vitro studies and cohort studies prompted questions on a possible adverse effect of insulin use on cardiovascular risk [13]. To further elucidate this association, we also included type 2 diabetes patients using insulin. These patients were characterized by a 1.5-fold further increase in ^18^F-FDG uptake compared with type 2 diabetes patients not using insulin. Given the worse regulation of diabetes in insulin users (Hba1c in PAD-IDDM 78 mmol/mol versus 49 mmol/mol in PAD-NIDDM), we cannot discriminate the role of exogenous insulin use versus worse diabetic control. However, daily insulin dose did not correlate with arterial ^18^F-FDG uptake, whereas plasma insulin concentrations and indices of insulin resistance were highly correlated with ^18^F-FDG-uptake. These findings imply that insulin resistance and hyperglycaemia, rather than the use of exogenous insulin is associated with inflammatory activation. In support, a recent analysis in the randomized, placebo-controlled ACCORD trial also could not substantiate the link between insulin dose used and CV events, after adjustment for baseline covariates [26].

### Limitations

Several limitations need to be taken into account when interpreting our data. First, previous studies in other diseases were available to underpin the sample size calculation for the comparison between PAD patients and controls, but no data were available to determine the appropriate sample size for the other analyses. As such, the other analyses may be underpowered and should be interpreted as merely hypothesis forming. Moreover, the study design precludes any conclusions on causality of the observed associations, and the different presence of other cardiovascular risk factors associated with diabetes and CVD complicates interpretation of our findings. For example hypertension, represented by the higher level of use of antihypertensive medication was more often present in the diabetes group, however, treated systolic blood pressure was not significantly associated with arterial inflammation [11]. Thus, our data contribute to a growing body of evidence highlighting the importance of inflammation in the adverse CVD risk profile of patients with diabetes and highlight the need to understand underlying pathophysiology.

Second, we did not evaluate arterial wall inflammation in other territories than the carotid arteries. The carotid artery has been recommended as a primary readout vessel to evaluate arterial wall inflammation because it has been most extensively validated. Thus, we considered the carotid artery as an appropriate measure to evaluate the extent of *systemic* arterial wall inflammation in PAD patients [16]. ^18^F-FDG PET/CT can be excellently reproduced across the arterial tree, as published previously [27]. We did not perform structural measurements of the carotid arteries (MRI or ultrasound) to assess carotid atherosclerotic burden. However, Tahara and colleagues previously established a correlation between carotid intima-media thickness (c-IMT) and SUV uptake [12] and as outlined in the introduction, arterial inflammation has been extensively confirmed as an independent measure for CVD risk. Finally, given the cross-sectional design of the current study, no conclusions can be drawn on the implications of the observed associations in terms of clinical outcomes. Although ^18^F-FDG has been shown to be of value in assessing cardiovascular risk [9], glucose-corrected TBRs have not been validated concerning definitive outcomes, and long term studies are necessary to prove its relation with CV-events.

## Conclusions

In the current study, we show that PAD patients have significantly increased inflammation in the carotid arteries, which is augmented by the presence of type 2 diabetes. Given the particularly adverse CVD risk profile of PAD patients with concomitant diabetes and the growing body of evidence on the importance of inflammation in both atherosclerosis and diabetes, our findings suggests that arterial wall inflammation is involved in the adverse CVD risk profile of these patients. Identification of key mediators may aid in more targeted prevention in the future.

## Additional file

Additional file 1: Table S1. Fontaine classification of PAD. **Table S2.** Linear regression analysis for insulin in diabetic subjects. **Table S3.** Medication use in PAD subjects. **Table S4.** Linear regression analysis for insulin in diabetic subjects. **Figure S1.** Target to Background ratio (TBR_mean_) did not correlate to plasma C-peptide levels. (PDF 53 kb)

## Abbreviations

^18^F-FDG: ^18^F-fluorodeoxyglucose
ABI: Ankle brachial index
BMI: Body mass index
CAD: Coronary artery disease
CRP: C-reactive protein
CT: Computed tomography
CVD: Cardiovascular disease
DBP: Diastolic blood pressure
FFA: Free fatty acid
HDL: High-density lipoprotein cholesterol
HOMA: Homeostasis model assessment
IDDM: Insulin dependent diabetes mellitus
LDL: Low-density lipoprotein cholesterol
MDS: Most-diseased segment
NIDDM: Non- insulin dependent diabetes mellitus
PAD: Peripheral artery disease
PET: Positron emission tomography
ROI: Regions of interest
SBP: Systolic blood pressure
SUV: Standardized uptake values
TBR: Target to background ratio
TChol: Total cholesterol
TG: Triglycerides
