# Supplementary material for: Carotid arterial wall inflammation in peripheral artery disease is augmented by type 2 diabetes: a cross-sectional study
Source: BMC Cardiovasc Disord. 2016 Nov 25;16:237. doi: 10.1186/s12872-016-0397-x (PMC5124245; doi:10.1186/s12872-016-0397-x)

Supplemental Material

Supplemental Methods

Power calculations

Our primary outcome will be the degree of arterial wall inflammation in patients with PAD, which we will compare to historical cohorts of healthy controls. Other studies have indicated that the mean maximal TBR in healthy controls is 1.6, whereas mean maximal TBR in CVD patients is 2.0. Moreover, in concordance with Rudd et al, we normally have a common standard deviation of 0.20 for vascular ^{18}F-FDG PET/CT imaging (Rudd, JACC 2007). Thus, when assuming a two group t-test with a 0,050 two-sided significance level and a power of 80%, we need to include 17 patients to observe a difference of at least 0.2 in TBR between groups.

Supplemental table S1: Overview of powercalculation

**Test significance level, a**	0.05
**1 or 2 sided test?**	2
**Difference in means, m_1 - m_2**	0.20
**Common standard deviation, s**	0.20
**Power (%)**	80
**n per group**	**17**

Supplemental table S2: Fontaine classification of PAD

	2a	2b	3	Total
**PAD only**	7	3	1	11
**PAD-DM**	13	6	4	23
**NIDDM**	6	3	2	11
**IDDM**	7	3	2	12

Data are n. PAD indicates Peripheral Artery Disease; *DM* Diabetes Mellitus; *NIDDM* Non-insulin dependent diabetes mellitus; *IDDM* insulin-dependent diabetes.

Supplemental table S3: Medication use in PAD subjects

Type of medication	PAD only n= 11	PAD-DM n= 23	P-value	PAD-NIDDM n= 11	PAD-IDDM n=12	P-value
Statin	10	19	0.532	8	11	0.231
Antihypertensive	6	22	0.003	11	11	0.382
ACI/ARB	4	14	0.180	7	7	0.795
Calcium antagonist	2	11	0.096	4	7	0.292
Diuretic	4	13	0.271	7	6	0.510
Beta blocker	4	9	0.877	4	5	0.795

Data are n. PAD indicates Peripheral Artery Disease; *DM* Diabetes Mellitus; *NIDDM* Non-insulin dependent diabetes mellitus; *IDDM* insulin-dependent diabetes; ACI angiotensin-converting-enzyme inhibitor; ARB angiotensin II receptor antagonist

Supplemental Table S4: Linear regression analysis for insulin in diabetic subjects

Variable	R square	P value
C-peptide	-0.037	0.538
Daily insulin dose	0.007	0.345

Insulin is the response variable. Explanatory factors comprised C-peptide and daily insulin dose.

Data are R square

Supplemental Figure legend

Supplemental Figure 1: Target to Background ratio (TBR_{mean}) did not correlate to plasma C-peptide levels (Correlation coefficient:, -0.019, p=0.936).

Supplemental Figure 1

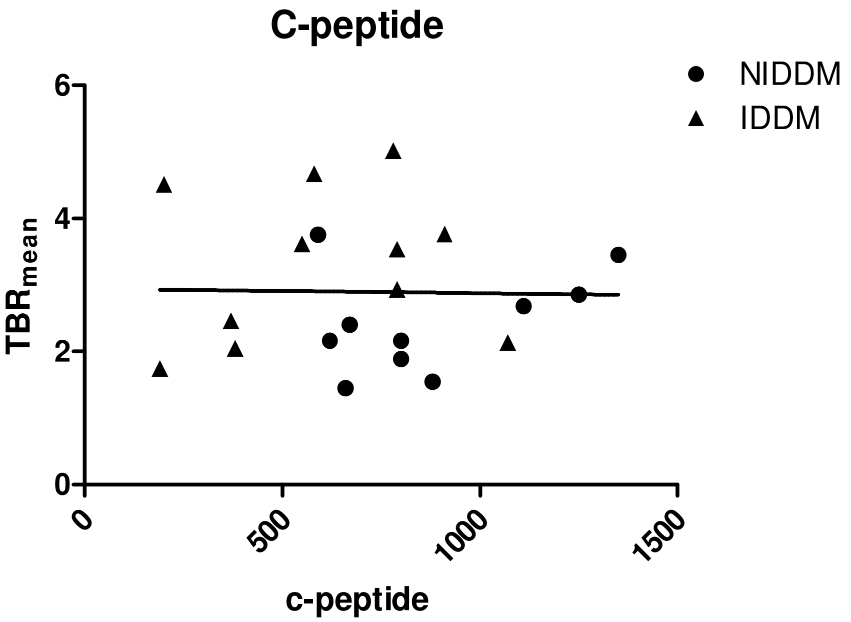

Supplement: Additional file 1: Table S1. — Fontaine classification of PAD. Table S2. Linear regression analysis for insulin in diabetic subjects. Table S3. Medication use in PAD subjects. Table S4. Linear regression analysis for insulin in diabetic subjects. Figure S1. Target to Background ratio (TBRmean) did not correlate to plasma C-peptide levels. (PDF 53 kb) [file 12872_2016_397_MOESM1_ESM.pdf]
